# *trans*-Bis(5,5-diphenyl­hydantoinato-κ*N* ^3^)bis­(propane-1,2-diamine-κ^2^ *N*,*N*′)nickel(II)

**DOI:** 10.1107/S1600536808038749

**Published:** 2008-11-29

**Authors:** Xilan Hu, Xingyou Xu, Daqi Wang, Xiaojiao Li

**Affiliations:** a Huaihai Institute of Technology, Jiangsu 222005, People’s Republic of China; b College of Chemistry and Chemical Engineering, Liaocheng University, Shandong 252059, People’s Republic of China

## Abstract

The asymmetric unit of the title complex, [Ni(pht)_2_(pn)_2_] (pht is 5,5-diphenyl­hydantoinate and pn is propane-1,2-diamine) or [Ni(C_15_H_11_N_2_O_2_)_2_(C_3_H_10_N_2_)_2_], contains one-half [Ni(pht)_2_(pn)_2_] mol­ecule. The Ni^II^ atom is situated on a crystallographic center of inversion and shows a distorted octa­hedral coordination geometry. A three-dimensional network structure is assembled by inter- and intra­molecular N—H⋯O=C inter­actions.

## Related literature

For general background see Akitsu *et al.* (1997 ▶), Milne *et al.* (1999 ▶). For related structures see Akitsu & Einaga *et al.* (2005 ▶); Hu *et al.* (2006*a* ▶,*b* ▶).
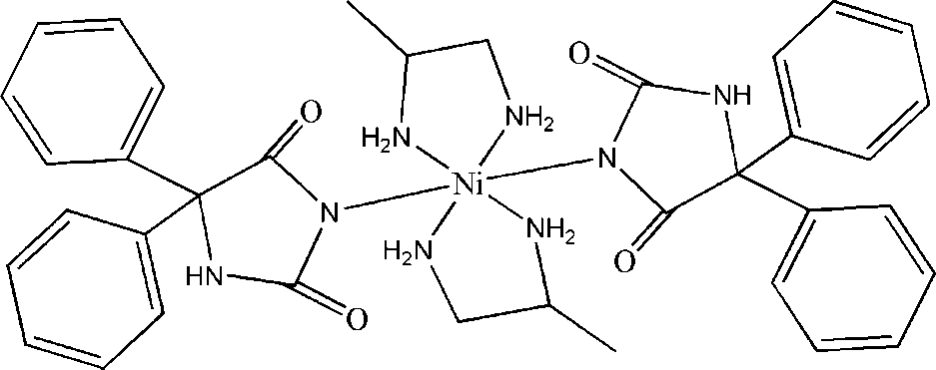


## Experimental

### 

#### Crystal data

[Ni(C_15_H_11_N_2_O_2_)_2_(C_3_H_10_N_2_)_2_]
*M* *_r_* = 709.49
Triclinic, 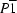

*a* = 8.581 (1) Å
*b* = 9.731 (1) Å
*c* = 12.036 (2) Å
α = 100.602 (2)°
β = 90.298 (1)°
γ = 113.951 (2)°
*V* = 899.2 (2) Å^3^
*Z* = 1
Mo *K*α radiation
μ = 0.59 mm^−1^
*T* = 298 (2) K
0.47 × 0.45 × 0.36 mm

#### Data collection

Bruker SMART CCD area-detector diffractometer
Absorption correction: multi-scan (*SADABS*; Sheldrick, 1996 ▶) *T*_min_ = 0.769, *T*_max_ = 0.816
4760 measured reflections
3164 independent reflections
2908 reflections with *I* > 2σ(*I*)
*R*_int_ = 0.011

#### Refinement

*R*[*F* ^2^ > 2σ(*F* ^2^)] = 0.028
*wR*(*F* ^2^) = 0.074
*S* = 1.09
3164 reflections
253 parameters
H-atom parameters constrained
Δρ_max_ = 0.17 e Å^−3^
Δρ_min_ = −0.22 e Å^−3^

### 

Data collection: *SMART* (Siemens, 1996 ▶); cell refinement: *SAINT* (Siemens, 1996 ▶); data reduction: *SAINT*; program(s) used to solve structure: *SHELXS97* (Sheldrick, 2008 ▶); program(s) used to refine structure: *SHELXL97* (Sheldrick, 2008 ▶); molecular graphics: *SHELXTL* (Sheldrick, 2008 ▶); software used to prepare material for publication: *SHELXTL*.

## Supplementary Material

Crystal structure: contains datablocks I, global. DOI: 10.1107/S1600536808038749/im2088sup1.cif

Structure factors: contains datablocks I. DOI: 10.1107/S1600536808038749/im2088Isup2.hkl

Additional supplementary materials:  crystallographic information; 3D view; checkCIF report

## Figures and Tables

**Table 1:** Hydrogen-bond geometry (Å, °)

*D*—H⋯*A*	*D*—H	H⋯*A*	*D*⋯*A*	*D*—H⋯*A*
N3—H3′*B*⋯O2	0.90	2.55	3.231 (2)	133
N4—H4*A*⋯O1	0.90	2.26	2.983 (2)	138

**Fig. 1.**
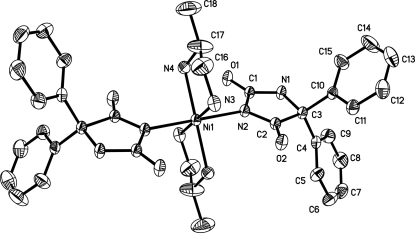
The molecular structure of the title complex. Displacement ellipsoids are drawn at the 30% probability level.

**Fig. 2.**
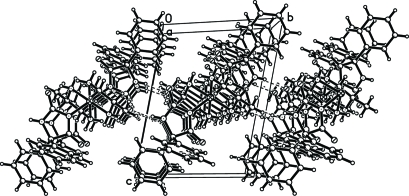
The crystal packing of the title complex.

## supplementary crystallographic information

### Comment

5,5-Diphenylimidazoline-2,4-dione (Hpht) is a widely used drug in the treatment of epilepsy. It should also be an excellent ligand for transition metal complexes (Milne *et al.*,1999; Akitsu *et al.*,1997; Akitsu & Einaga, 2005). We have therefore designed and synthesized a series of complexes with 5,5-diphenylhydantoinato ligands (Hu *et al.*, 2006a).

The title compound (Fig. 1) consists of a neutral [Ni(pht)_2_(pn)_2_] complex molecule. The nickel atom is situated at the crystallographic center of inversion and is coordinated by two nitrogen atoms from two pht ligands and four nitrogen atoms from two pn ligands. The metal atom therefore adopts a distorted octahedral NiN_6_ coordination environment with a dihedral angle of 86.9 (1)° between N3—N3A—N4A—N4 and the hydantoin ring and dihedral angles between N3—N3A—N4A—N4 and the pht groups of 51.7 (1)° (C4 to C9) and 39.0 (1)° (C10 to C15), respectively. The Ni—N bond distances lie in the range of 2.096 (2) Å to 2.125 (2) Å. Intramolecular hydrogen bonds (Table 1) serve to stabilize the octahedral geometry. Adjacent molecules are linked by intermolecular hydrogen bonds along the crystallographic *a* axis. A similar hydrogen-bonding pattern is also found in the above-mentioned related complexes. The complex shows a three-dimensional network structure assembled by additional intermolecular N—H···O hydrogen bonds between the diamine ligand and the hydantoin ring.

### Experimental

To a solution of Hpht (1.00 mmol) in methanol (10 ml) was added Ni(OAc)_2_ × 4 H_2_O (0.5 mmol) and a solution of propane-1,2-diamine (1 mmol) in methanol (10 ml). Then the mixture was sealed in a 25 ml PTFE-lined stainless steel autoclave and heated to 423 K for 40 h, the fill rate being 80%. After cooling to room temperature, purple single crystals of the title compound were obtained by slow evaporation from the filtrate. Analysis, calculated for C_36_H_42_N_8_NiO_4_: C 61.66, H 6.01, N 16.26; found: C 60.94, H 5.97, N 15.78%.

### Refinement

The space group was assigned from the systematic absences. All H atoms were placed at calculated positions, with N—H = 0.86–0.89 Å and *U*_iso_(H) values of 1.2*U*_eq_(N), and C—H = 0.96 Å (methyl), 0.97 Å (methylene), 0.98 Å (methyne) and 0.93 Å (aryl), respectively, with *U*_iso_(H) values of 1.2 *U*_eq_(C) (methylene, methyne, aryl) or 1.5 *U*_eq_(C) (methyl).

### Crystal data

**Table:**

[Ni(C_15_H_11_N_2_O_2_)_2_(C_3_H_10_N_2_)_2_]	*Z* = 1
*M**_r_* = 709.49	*F*_000_ = 374
Triclinic, *P*1	*D*_x_ = 1.310 Mg m^−^^3^
Hall symbol: -P 1	Mo *K*α radiation λ = 0.71073 Å
*a* = 8.581 (1) Å	Cell parameters from 3393 reflections
*b* = 9.731 (1) Å	θ = 2.6–28.2º
*c* = 12.036 (2) Å	µ = 0.59 mm^−^^1^
α = 100.602 (2)º	*T* = 298 (2) K
β = 90.298 (1)º	Block, violet
γ = 113.951 (2)º	0.47 × 0.45 × 0.36 mm
*V* = 899.2 (2) Å^3^	

### Data collection

**Table:**

Bruker SMART CCD area-detector diffractometer	3164 independent reflections
Radiation source: fine-focus sealed tube	2908 reflections with *I* > 2σ(*I*)
Monochromator: graphite	*R*_int_ = 0.011
*T* = 298(2) K	θ_max_ = 25.0º
φ and ω scans	θ_min_ = 1.7º
Absorption correction: multi-scan(SADABS; Sheldrick, 1996)	*h* = −7→10
*T*_min_ = 0.769, *T*_max_ = 0.816	*k* = −11→11
4760 measured reflections	*l* = −14→14

### Refinement

**Table:**

Refinement on *F*^2^	Secondary atom site location: difference Fourier map
Least-squares matrix: full	Hydrogen site location: inferred from neighbouring sites
*R*[*F*^2^ > 2σ(*F*^2^)] = 0.028	H-atom parameters constrained
*wR*(*F*^2^) = 0.075	*w* = 1/[σ^2^(*F*_o_^2^) + (0.0351*P*)^2^ + 0.2523*P*] where *P* = (*F*_o_^2^ + 2*F*_c_^2^)/3
*S* = 1.09	(Δ/σ)_max_ = 0.001
3164 reflections	Δρ_max_ = 0.17 e Å^−^^3^
253 parameters	Δρ_min_ = −0.22 e Å^−^^3^
Primary atom site location: structure-invariant direct methods	Extinction correction: none

### Special details

**Table:**

Geometry. All e.s.d.'s (except the e.s.d. in the dihedral angle between two l.s. planes) are estimated using the full covariance matrix. The cell e.s.d.'s are taken into account individually in the estimation of e.s.d.'s in distances, angles and torsion angles; correlations between e.s.d.'s in cell parameters are only used when they are defined by crystal symmetry. An approximate (isotropic) treatment of cell e.s.d.'s is used for estimating e.s.d.'s involving l.s. planes.
Refinement. Refinement of *F*^2^ against ALL reflections. The weighted *R*-factor *wR* and goodness of fit *S* are based on *F*^2^, conventional *R*-factors *R* are based on *F*, with *F* set to zero for negative *F*^2^. The threshold expression of *F*^2^ > σ(*F*^2^) is used only for calculating *R*-factors(gt) *etc*. and is not relevant to the choice of reflections for refinement. *R*-factors based on *F*^2^ are statistically about twice as large as those based on *F*, and *R*- factors based on ALL data will be even larger.

### Fractional atomic coordinates and isotropic or equivalent isotropic displacement parameters (Å^2^)

**Table:**

	*x*	*y*	*z*	*U*_iso_*/*U*_eq_	Occ. (<1)
Ni1	0.5000	0.5000	0.5000	0.02926 (10)	
N1	0.09328 (19)	0.14829 (16)	0.62697 (11)	0.0390 (4)	
H1	0.0097	0.0587	0.6140	0.047*	
N2	0.31918 (17)	0.35330 (15)	0.59323 (11)	0.0327 (3)	
N3	0.70216 (19)	0.52417 (18)	0.61129 (13)	0.0448 (4)	
H3A	0.7766	0.6235	0.6302	0.054*	0.749 (12)
H3B	0.6617	0.4923	0.6751	0.054*	0.749 (12)
H3'A	0.7990	0.6047	0.6039	0.054*	0.251 (12)
H3'B	0.6766	0.5364	0.6839	0.054*	0.251 (12)
N4	0.5406 (2)	0.31168 (17)	0.41315 (13)	0.0426 (4)	
H4A	0.4402	0.2291	0.3932	0.051*	0.749 (12)
H4B	0.5927	0.3333	0.3499	0.051*	0.749 (12)
H4'A	0.4581	0.2250	0.4272	0.051*	0.251 (12)
H4'B	0.5306	0.3081	0.3381	0.051*	0.251 (12)
O1	0.17051 (16)	0.14829 (14)	0.44533 (10)	0.0465 (3)	
O2	0.39713 (17)	0.50006 (14)	0.77351 (10)	0.0473 (3)	
C1	0.1917 (2)	0.21085 (18)	0.54757 (13)	0.0337 (4)	
C2	0.3030 (2)	0.38406 (18)	0.70541 (13)	0.0329 (4)	
C3	0.1457 (2)	0.25095 (18)	0.73787 (13)	0.0330 (4)	
C4	0.0040 (2)	0.3011 (2)	0.77944 (14)	0.0368 (4)	
C5	0.0355 (3)	0.4505 (2)	0.82802 (17)	0.0510 (5)	
H5	0.1464	0.5265	0.8351	0.061*	
C6	−0.0972 (3)	0.4891 (3)	0.86675 (19)	0.0635 (6)	
H6	−0.0740	0.5905	0.8991	0.076*	
C7	−0.2594 (3)	0.3800 (3)	0.85770 (19)	0.0650 (6)	
H7	−0.3475	0.4064	0.8836	0.078*	
C8	−0.2934 (3)	0.2302 (3)	0.8102 (2)	0.0712 (7)	
H8	−0.4047	0.1551	0.8043	0.085*	
C9	−0.1620 (3)	0.1901 (3)	0.7708 (2)	0.0582 (5)	
H9	−0.1861	0.0884	0.7385	0.070*	
C10	0.1974 (2)	0.1815 (2)	0.82832 (14)	0.0370 (4)	
C11	0.2842 (3)	0.2747 (2)	0.93080 (16)	0.0503 (5)	
H11	0.3119	0.3795	0.9434	0.060*	
C12	0.3302 (3)	0.2147 (3)	1.01427 (19)	0.0684 (6)	
H12	0.3890	0.2790	1.0822	0.082*	
C13	0.2896 (4)	0.0616 (4)	0.9973 (2)	0.0843 (8)	
H13	0.3212	0.0211	1.0533	0.101*	
C14	0.2023 (4)	−0.0325 (3)	0.8978 (3)	0.0929 (9)	
H14	0.1736	−0.1373	0.8866	0.112*	
C15	0.1555 (3)	0.0270 (2)	0.8124 (2)	0.0637 (6)	
H15	0.0961	−0.0381	0.7450	0.076*	
C16	0.7858 (7)	0.4340 (8)	0.5562 (4)	0.0575 (12)	0.749 (12)
H16A	0.8554	0.4180	0.6118	0.069*	0.749 (12)
H16B	0.8602	0.4873	0.5032	0.069*	0.749 (12)
C17	0.6525 (7)	0.2805 (5)	0.4932 (5)	0.0575 (12)	0.749 (12)
H17	0.5812	0.2275	0.5487	0.069*	0.749 (12)
C18	0.7400 (14)	0.1808 (15)	0.4340 (10)	0.099 (3)	0.749 (12)
H18A	0.6551	0.0791	0.4038	0.148*	0.749 (12)
H18B	0.8197	0.1759	0.4877	0.148*	0.749 (12)
H18C	0.7999	0.2251	0.3734	0.148*	0.749 (12)
C16'	0.722 (2)	0.368 (2)	0.5724 (13)	0.056 (4)	0.251 (12)
H16C	0.8332	0.3810	0.6020	0.068*	0.251 (12)
H16D	0.6356	0.2892	0.6048	0.068*	0.251 (12)
C17'	0.7033 (17)	0.3156 (18)	0.4444 (14)	0.060 (3)	0.251 (12)
H17'	0.7959	0.3857	0.4081	0.072*	0.251 (12)
C18'	0.683 (4)	0.149 (5)	0.410 (3)	0.103 (8)	0.251 (12)
H18D	0.5901	0.0839	0.4465	0.154*	0.251 (12)
H18E	0.7867	0.1422	0.4322	0.154*	0.251 (12)
H18F	0.6582	0.1151	0.3289	0.154*	0.251 (12)

### Atomic displacement parameters (Å^2^)

**Table:**

	*U*^11^	*U*^22^	*U*^33^	*U*^12^	*U*^13^	*U*^23^
Ni1	0.02461 (16)	0.02656 (16)	0.02889 (16)	0.00303 (12)	0.00163 (11)	0.00520 (11)
N1	0.0362 (8)	0.0296 (7)	0.0283 (7)	−0.0070 (6)	0.0060 (6)	0.0005 (6)
N2	0.0296 (7)	0.0271 (7)	0.0285 (7)	−0.0002 (6)	0.0031 (6)	0.0033 (5)
N3	0.0354 (8)	0.0493 (9)	0.0422 (8)	0.0079 (7)	−0.0019 (7)	0.0143 (7)
N4	0.0407 (9)	0.0342 (8)	0.0462 (9)	0.0099 (7)	0.0075 (7)	0.0059 (7)
O1	0.0454 (8)	0.0378 (7)	0.0282 (6)	−0.0079 (6)	0.0059 (5)	−0.0010 (5)
O2	0.0453 (7)	0.0366 (7)	0.0329 (6)	−0.0059 (6)	0.0031 (6)	−0.0030 (5)
C1	0.0311 (9)	0.0292 (8)	0.0301 (8)	0.0024 (7)	0.0034 (7)	0.0046 (7)
C2	0.0304 (9)	0.0290 (8)	0.0304 (8)	0.0039 (7)	0.0031 (7)	0.0048 (7)
C3	0.0313 (9)	0.0295 (8)	0.0272 (8)	0.0025 (7)	0.0038 (7)	0.0032 (6)
C4	0.0355 (9)	0.0441 (10)	0.0297 (8)	0.0134 (8)	0.0061 (7)	0.0119 (7)
C5	0.0467 (11)	0.0520 (12)	0.0472 (11)	0.0180 (10)	0.0040 (9)	−0.0008 (9)
C6	0.0677 (16)	0.0705 (15)	0.0572 (13)	0.0382 (13)	0.0098 (11)	0.0017 (11)
C7	0.0620 (15)	0.0929 (19)	0.0575 (13)	0.0471 (14)	0.0196 (11)	0.0201 (13)
C8	0.0395 (12)	0.0841 (18)	0.0908 (18)	0.0206 (12)	0.0208 (12)	0.0308 (15)
C9	0.0409 (11)	0.0533 (12)	0.0777 (15)	0.0136 (10)	0.0168 (11)	0.0209 (11)
C10	0.0328 (9)	0.0395 (9)	0.0354 (9)	0.0107 (8)	0.0108 (7)	0.0102 (7)
C11	0.0544 (12)	0.0543 (12)	0.0382 (10)	0.0192 (10)	0.0011 (9)	0.0078 (9)
C12	0.0703 (16)	0.0855 (18)	0.0460 (12)	0.0272 (14)	−0.0034 (11)	0.0173 (12)
C13	0.099 (2)	0.096 (2)	0.0688 (17)	0.0404 (18)	−0.0025 (15)	0.0412 (16)
C14	0.125 (3)	0.0590 (16)	0.100 (2)	0.0353 (17)	0.000 (2)	0.0359 (16)
C15	0.0815 (17)	0.0425 (12)	0.0592 (13)	0.0169 (11)	−0.0027 (12)	0.0126 (10)
C16	0.041 (2)	0.068 (3)	0.067 (2)	0.024 (2)	−0.0031 (18)	0.021 (2)
C17	0.064 (3)	0.054 (2)	0.067 (3)	0.036 (2)	0.009 (2)	0.0153 (19)
C18	0.106 (7)	0.102 (6)	0.111 (5)	0.081 (6)	−0.013 (4)	−0.014 (4)
C16'	0.053 (9)	0.054 (8)	0.071 (8)	0.031 (7)	−0.006 (6)	0.014 (7)
C17'	0.051 (6)	0.080 (8)	0.064 (8)	0.039 (6)	0.019 (5)	0.021 (6)
C18'	0.085 (16)	0.099 (18)	0.14 (2)	0.063 (15)	−0.022 (13)	0.007 (15)

### Geometric parameters (Å, °)

**Table:**

Ni1—N3^i^	2.0946 (15)	C6—H6	0.9300
Ni1—N3	2.0946 (15)	C7—C8	1.371 (4)
Ni1—N4	2.0950 (15)	C7—H7	0.9300
Ni1—N4^i^	2.0950 (15)	C8—C9	1.394 (3)
Ni1—N2^i^	2.1245 (13)	C8—H8	0.9300
Ni1—N2	2.1245 (13)	C9—H9	0.9300
N1—C1	1.343 (2)	C10—C15	1.372 (3)
N1—C3	1.456 (2)	C10—C11	1.389 (3)
N1—H1	0.8600	C11—C12	1.380 (3)
N2—C2	1.349 (2)	C11—H11	0.9300
N2—C1	1.379 (2)	C12—C13	1.359 (4)
N3—C16	1.423 (5)	C12—H12	0.9300
N3—C16'	1.580 (13)	C13—C14	1.366 (4)
N3—H3A	0.9000	C13—H13	0.9300
N3—H3B	0.9000	C14—C15	1.398 (3)
N3—H3'A	0.9000	C14—H14	0.9300
N3—H3'B	0.9000	C15—H15	0.9300
N4—C17'	1.428 (11)	C16—C17	1.517 (8)
N4—C17	1.510 (4)	C16—H16A	0.9700
N4—H4A	0.9000	C16—H16B	0.9700
N4—H4B	0.9000	C17—C18	1.534 (11)
N4—H4'A	0.9000	C17—H17	0.9800
N4—H4'B	0.9000	C18—H18A	0.9600
O1—C1	1.245 (2)	C18—H18B	0.9600
O2—C2	1.2292 (19)	C18—H18C	0.9600
C2—C3	1.557 (2)	C16'—C17'	1.52 (3)
C3—C10	1.532 (2)	C16'—H16C	0.9700
C3—C4	1.538 (2)	C16'—H16D	0.9700
C4—C5	1.377 (3)	C17'—C18'	1.54 (4)
C4—C9	1.383 (3)	C17'—H17'	0.9800
C5—C6	1.393 (3)	C18'—H18D	0.9600
C5—H5	0.9300	C18'—H18E	0.9600
C6—C7	1.354 (3)	C18'—H18F	0.9600
			
N3^i^—Ni1—N3	180.000 (1)	C10—C3—C4	109.20 (13)
N3^i^—Ni1—N4	96.99 (6)	N1—C3—C2	98.65 (12)
N3—Ni1—N4	83.01 (6)	C10—C3—C2	111.63 (14)
N3^i^—Ni1—N4^i^	83.01 (6)	C4—C3—C2	112.68 (14)
N3—Ni1—N4^i^	96.99 (6)	C5—C4—C9	118.46 (18)
N4—Ni1—N4^i^	180.00 (8)	C5—C4—C3	123.02 (16)
N3^i^—Ni1—N2^i^	90.86 (6)	C9—C4—C3	118.48 (17)
N3—Ni1—N2^i^	89.14 (6)	C4—C5—C6	120.7 (2)
N4—Ni1—N2^i^	90.56 (6)	C4—C5—H5	119.7
N4^i^—Ni1—N2^i^	89.44 (6)	C6—C5—H5	119.7
N3^i^—Ni1—N2	89.14 (6)	C7—C6—C5	120.5 (2)
N3—Ni1—N2	90.86 (6)	C7—C6—H6	119.7
N4—Ni1—N2	89.44 (6)	C5—C6—H6	119.7
N4^i^—Ni1—N2	90.56 (6)	C6—C7—C8	119.7 (2)
N2^i^—Ni1—N2	180.00 (7)	C6—C7—H7	120.1
C1—N1—C3	111.57 (13)	C8—C7—H7	120.1
C1—N1—H1	124.2	C7—C8—C9	120.3 (2)
C3—N1—H1	124.2	C7—C8—H8	119.8
C2—N2—C1	107.79 (13)	C9—C8—H8	119.8
C2—N2—Ni1	126.79 (11)	C4—C9—C8	120.2 (2)
C1—N2—Ni1	125.37 (10)	C4—C9—H9	119.9
C16—N3—C16'	26.9 (6)	C8—C9—H9	119.9
C16—N3—Ni1	108.5 (2)	C15—C10—C11	118.31 (18)
C16'—N3—Ni1	103.2 (5)	C15—C10—C3	121.58 (17)
C16—N3—H3A	110.0	C11—C10—C3	120.09 (16)
C16'—N3—H3A	134.1	C12—C11—C10	121.2 (2)
Ni1—N3—H3A	110.0	C12—C11—H11	119.4
C16—N3—H3B	110.0	C10—C11—H11	119.4
C16'—N3—H3B	88.1	C13—C12—C11	120.0 (2)
Ni1—N3—H3B	110.0	C13—C12—H12	120.0
H3A—N3—H3B	108.4	C11—C12—H12	120.0
C16—N3—H3'A	84.6	C12—C13—C14	119.7 (2)
C16'—N3—H3'A	110.8	C12—C13—H13	120.1
Ni1—N3—H3'A	111.2	C14—C13—H13	120.1
H3A—N3—H3'A	27.1	C13—C14—C15	120.8 (2)
H3B—N3—H3'A	128.4	C13—C14—H14	119.6
C16—N3—H3'B	128.8	C15—C14—H14	119.6
C16'—N3—H3'B	111.3	C10—C15—C14	119.9 (2)
Ni1—N3—H3'B	111.2	C10—C15—H15	120.0
H3A—N3—H3'B	85.3	C14—C15—H15	120.0
H3B—N3—H3'B	24.9	N3—C16—C17	109.4 (4)
H3'A—N3—H3'B	109.1	N3—C16—H16A	109.8
C17'—N4—C17	30.6 (6)	C17—C16—H16A	109.8
C17'—N4—Ni1	113.6 (5)	N3—C16—H16B	109.8
C17—N4—Ni1	106.75 (19)	C17—C16—H16B	109.8
C17'—N4—H4A	128.2	H16A—C16—H16B	108.2
C17—N4—H4A	110.4	N4—C17—C16	107.8 (4)
Ni1—N4—H4A	110.4	N4—C17—C18	113.7 (5)
C17'—N4—H4B	80.3	C16—C17—C18	110.2 (6)
C17—N4—H4B	110.4	N4—C17—H17	108.3
Ni1—N4—H4B	110.4	C16—C17—H17	108.3
H4A—N4—H4B	108.6	C18—C17—H17	108.3
C17'—N4—H4'A	108.7	C17'—C16'—N3	113.0 (12)
C17—N4—H4'A	84.0	C17'—C16'—H16C	109.0
Ni1—N4—H4'A	108.7	N3—C16'—H16C	109.0
H4A—N4—H4'A	29.0	C17'—C16'—H16D	109.0
H4B—N4—H4'A	131.5	N3—C16'—H16D	109.0
C17'—N4—H4'B	109.2	H16C—C16'—H16D	107.8
C17—N4—H4'B	136.1	N4—C17'—C16'	102.5 (12)
Ni1—N4—H4'B	108.9	N4—C17'—C18'	105.2 (14)
H4A—N4—H4'B	80.1	C16'—C17'—C18'	112 (2)
H4B—N4—H4'B	31.9	N4—C17'—H17'	112.3
H4'A—N4—H4'B	107.6	C16'—C17'—H17'	112.3
O1—C1—N1	124.25 (15)	C18'—C17'—H17'	112.3
O1—C1—N2	124.13 (14)	C17'—C18'—H18D	109.5
N1—C1—N2	111.62 (13)	C17'—C18'—H18E	109.5
O2—C2—N2	125.70 (15)	H18D—C18'—H18E	109.5
O2—C2—C3	124.01 (14)	C17'—C18'—H18F	109.5
N2—C2—C3	110.29 (13)	H18D—C18'—H18F	109.5
N1—C3—C10	113.28 (14)	H18E—C18'—H18F	109.5
N1—C3—C4	111.15 (14)		
			
N3^i^—Ni1—N2—C2	−125.79 (15)	O2—C2—C3—C4	65.1 (2)
N3—Ni1—N2—C2	54.21 (15)	N2—C2—C3—C4	−114.64 (15)
N4—Ni1—N2—C2	137.22 (15)	N1—C3—C4—C5	−135.47 (17)
N4^i^—Ni1—N2—C2	−42.78 (15)	C10—C3—C4—C5	98.84 (19)
N2^i^—Ni1—N2—C2	4(100)	C2—C3—C4—C5	−25.8 (2)
N3^i^—Ni1—N2—C1	51.22 (14)	N1—C3—C4—C9	46.6 (2)
N3—Ni1—N2—C1	−128.78 (14)	C10—C3—C4—C9	−79.1 (2)
N4—Ni1—N2—C1	−45.78 (14)	C2—C3—C4—C9	156.21 (17)
N4^i^—Ni1—N2—C1	134.22 (14)	C9—C4—C5—C6	−0.5 (3)
N2^i^—Ni1—N2—C1	−179 (100)	C3—C4—C5—C6	−178.45 (18)
N3^i^—Ni1—N3—C16	122 (100)	C4—C5—C6—C7	0.3 (3)
N4—Ni1—N3—C16	15.4 (3)	C5—C6—C7—C8	0.1 (4)
N4^i^—Ni1—N3—C16	−164.6 (3)	C6—C7—C8—C9	−0.4 (4)
N2^i^—Ni1—N3—C16	−75.2 (3)	C5—C4—C9—C8	0.3 (3)
N2—Ni1—N3—C16	104.8 (3)	C3—C4—C9—C8	178.32 (19)
N3^i^—Ni1—N3—C16'	95 (100)	C7—C8—C9—C4	0.2 (4)
N4—Ni1—N3—C16'	−12.0 (8)	N1—C3—C10—C15	−15.7 (2)
N4^i^—Ni1—N3—C16'	168.0 (8)	C4—C3—C10—C15	108.7 (2)
N2^i^—Ni1—N3—C16'	−102.7 (8)	C2—C3—C10—C15	−126.00 (19)
N2—Ni1—N3—C16'	77.3 (8)	N1—C3—C10—C11	166.25 (16)
N3^i^—Ni1—N4—C17'	162.2 (9)	C4—C3—C10—C11	−69.3 (2)
N3—Ni1—N4—C17'	−17.8 (9)	C2—C3—C10—C11	56.0 (2)
N4^i^—Ni1—N4—C17'	−110 (100)	C15—C10—C11—C12	1.0 (3)
N2^i^—Ni1—N4—C17'	71.3 (9)	C3—C10—C11—C12	179.14 (19)
N2—Ni1—N4—C17'	−108.7 (9)	C10—C11—C12—C13	−0.4 (4)
N3^i^—Ni1—N4—C17	−165.9 (3)	C11—C12—C13—C14	−0.4 (5)
N3—Ni1—N4—C17	14.1 (3)	C12—C13—C14—C15	0.7 (5)
N4^i^—Ni1—N4—C17	−78 (100)	C11—C10—C15—C14	−0.8 (4)
N2^i^—Ni1—N4—C17	103.1 (3)	C3—C10—C15—C14	−178.9 (2)
N2—Ni1—N4—C17	−76.9 (3)	C13—C14—C15—C10	0.0 (5)
C3—N1—C1—O1	−177.93 (17)	C16'—N3—C16—C17	40.6 (13)
C3—N1—C1—N2	1.9 (2)	Ni1—N3—C16—C17	−42.2 (6)
C2—N2—C1—O1	179.86 (17)	C17'—N4—C17—C16	68.4 (11)
Ni1—N2—C1—O1	2.4 (3)	Ni1—N4—C17—C16	−39.8 (6)
C2—N2—C1—N1	0.0 (2)	C17'—N4—C17—C18	−54.1 (13)
Ni1—N2—C1—N1	−177.47 (11)	Ni1—N4—C17—C18	−162.3 (7)
C1—N2—C2—O2	178.50 (18)	N3—C16—C17—N4	55.9 (7)
Ni1—N2—C2—O2	−4.1 (3)	N3—C16—C17—C18	−179.5 (6)
C1—N2—C2—C3	−1.79 (19)	C16—N3—C16'—C17'	−64.1 (18)
Ni1—N2—C2—C3	175.64 (10)	Ni1—N3—C16'—C17'	40.7 (17)
C1—N1—C3—C10	−120.79 (16)	C17—N4—C17'—C16'	−40.9 (13)
C1—N1—C3—C4	115.83 (16)	Ni1—N4—C17'—C16'	42.2 (18)
C1—N1—C3—C2	−2.67 (18)	C17—N4—C17'—C18'	76 (2)
O2—C2—C3—N1	−177.60 (17)	Ni1—N4—C17'—C18'	159.0 (19)
N2—C2—C3—N1	2.68 (18)	N3—C16'—C17'—N4	−55 (2)
O2—C2—C3—C10	−58.2 (2)	N3—C16'—C17'—C18'	−167.3 (14)
N2—C2—C3—C10	122.05 (15)		

Symmetry codes: (i) −*x*+1, −*y*+1, −*z*+1.

### Hydrogen-bond geometry (Å, °)

**Table:**

*D*—H···*A*	*D*—H	H···*A*	*D*···*A*	*D*—H···*A*
N3—H3'B···O2	0.90	2.55	3.231 (2)	133
N4—H4A···O1	0.90	2.26	2.983 (2)	138
